# Development and psychometric evaluation of the CanSmart questionnaire to measure chronic disease self-management tasks

**DOI:** 10.1186/s40359-022-00995-2

**Published:** 2022-12-07

**Authors:** Sylvie D. Lambert, Susan J. Bartlett, Jane McCusker, Mark Yaffe, Antonio Ciampi, Eric Belzile, Manon de Raad

**Affiliations:** 1 Ingram School of Nursing, McGill University, 680 Sherbrooke Ouest, #1800, Montreal, QC H3A 2M7 Canada; 2 Divisions of Clinical Epidemiology, Rheumatology and Respiratory Epidemiology and Clinical Trials Unit, Department of Medicine, McGill University, 1001 Decarie Boulevard, Montreal, QC H4A 3J1 Canada; 3 McGill University Health Center Research Institute, 5252 Boul. de Maisonneuve Ouest, Montreal, QC H4A 3S5 Canada; 4 St. Mary’s Research Centre, Hayes Pavilion, Suite 3734, 3830 Avenue Lacombe, Montreal, QC H3T 1M5 Canada; 5 Department of Epidemiology, Biostatistics and Occupational Health, McGill University, 942 Avenue des Pins Ouest, Montreal, H3A 1A2 Canada; 6 Department of Family Medicine, McGill University, 5858 Chemin de la Côte-des-Neiges, Montreal, H3S 1Z1 Canada; 7 Family Medicine Centre, St. Mary’s Hospital Center, 3830 Avenue Lacombe, Montreal, H3T 1M5 Canada

**Keywords:** Chronic disease, Self-management, Psychometric

## Abstract

**Background:**

Psychometrically sound measures of chronic disease self-management tasks are needed to improve identification of patient needs and to tailor self-management programs. This study aimed to develop and conduct a preliminary psychometric analysis of the CanSMART questionnaire among a diverse, multimorbid Canadian population.

**Methods:**

The data were drawn from a cross-sectional online survey to examine self-management needs and support preferences. Participants were 306 Canadian adults with one or more physical and/or emotional chronic conditions. The questionnaire on frequency of self-management tasks was developed with substantial patient partner input. We conducted Exploratory Factor Analysis (EFA) and Confirmatory Factor Analysis (CFA) of the 11 self-management tasks comprising the scale in two randomly selected subsamples, followed by Rasch analysis. Associations between patient characteristics and the self-management task subscales and individual items were explored.

**Results:**

The factor analyses identified two self-management task subscales that were labelled Coping tasks (6 items) and Physical tasks (3 items), with Cronbach’s alpha of 0.70 and 0.67, respectively. Rasch analysis suggested that participants had difficulty discriminating between response options “mostly” and “always”. In analyses of independent associations with patient characteristics, both Coping and Physical tasks were associated with reporting more than one chronic disease and employment disability. The Coping tasks subscale was associated with female sex. Two items, on medication use and monitoring biological parameters, did not load on either scale. Both were associated with specific diagnoses.

**Conclusions:**

In this preliminary analysis, two self-management tasks subscales exhibit good psychometric properties. Two items that did not load on either scale may represent additional dimensions of self-management. This work provides the basis for further scale development and use in research and clinical practice.

**Supplementary Information:**

The online version contains supplementary material available at 10.1186/s40359-022-00995-2.

## Introduction

The prevalence of chronic disease in Canada continues to grow rapidly, with over half of all Canadian adults already living with at least one chronic disease and 1 in 4 with multiple chronic diseases (multimorbidity) [1, 2]. These individuals often require ongoing, complex care and treatment and will experience effects on their physical and emotional quality of life [3, 4, 5]. The Chronic Care Model, involving pro-active healthcare providers and patients who are equipped to interact effectively with their healthcare team [6], changes the way chronic disease care is organized and delivered, leading to improved patient outcomes [7]. Self-management, a key component of the Chronic Care Model [6], refers to those behaviors necessary to promote one’s health and manage the physical, emotional, and social effects of an illness or illnesses [8]. People skilled at self-management understand their conditions and treatments and are actively involved in their care. They help create and follow their care plans, and monitor their conditions. Although self-management behaviors may vary by chronic disease, there may be core behaviors across diseases that are related in part to individual characteristics such as socio-economic status [9]. There is also generally good consensus from several major initiatives that there are common elements in chronic disease self-management, even across different chronic diseases [10, 11, 12, 13].

Whereas there are disease specific measures of self-management tasks (e.g., Diabetes Self-Management Questionnaire [14]), given the increasing prevalence of multimorbidity, valid and reliable measures applicable across chronic diseases to better understand all the self-management undertaken by patients are needed. Such measures would help to guide self-management support programs and tailor them to individual needs. Two previous studies have proposed categories of self-management tasks, but have not validated them. Schulman-Green [15] undertook a synthesis of 101 qualitative studies that described processes of self-management in chronic disease. These were summarized across three categories: (1) focusing on illness needs (including medication management, health behaviors, keeping appointments); (2) activating resources (including family, community, and healthcare resources) and living with a chronic illness (including coping skills such as processing emotions; and (3) adjusting to, and integrating illness into daily life. The second initiative, the Dutch Patient Assessment of Self-management Tasks (PAST) questionnaire [16], created utilizing expert input, included four categories of activities: (1) medical management, (2) communication with healthcare providers, (3) coping with the consequences of having a chronic illness, and (4) making lifestyle changes.

We sought to develop a psychometrically sound questionnaire on self-management task frequency that could be used in the Canadian context across chronic diseases and those with multimorbidity, inspired by the PAST, but using feedback from Canadian patients and self-management researchers and clinicians (CanSMART: Canadian Self-Management Research Team). The first version of the CanSMART self-management task questionnaire was used in an online survey of Canadian adults with chronic diseases [9]. In this paper, we report on the psychometric properties of this questionnaire.

## Methods

### Questionnaire development

Beginning in April 2015 CanSMART expert members collaborated to design an online survey to capture the self-management tasks, needs and support preferences of Canadians with a chronic illness. The CanSMART team was composed of 15 individuals from across Canada, including chronic disease researchers, clinical psychologists, family physicians, nurses, rehabilitation science practitioners, epidemiologists, and patient research partners. These stakeholders had come together as part of the Canadian Institutes of Health Research’s Strategy for Patient Oriented Research. Four successive versions of the survey design were circulated electronically for comments to the CanSMART team. Feedback was incorporated in turn, with each survey version discussed and further revised during team meetings until agreement among team members was reached.

Questions on self-management tasks were inspired by the structure of the PAST [16]. The PAST was developed in the Netherlands and asks respondents to indicate how often each of 19 listed self-management tasks are completed and how often support is needed with the tasks. Patient research partners first reviewed the wording and content of PAST items to assess their relevance to Canadians with chronic conditions. Additional input from other team members led to further changes: coping with pain and limitations was separated into 2 questions; 5 individual lifestyle questions on diet, exercise and health habits were collapsed into a single question; and new items were added to address asking for or needing help with household chores, self-care (e.g., bathing, dressing), and employment issues. The PAST items on interactions with healthcare providers (an important aspect of self-management) were excluded from the CanSMART questionnaire because corresponding questions from the Patient Assessment of Chronic Illness Care (PACIC) [17] were included in the larger survey [18]. Frequency of tasks was reported on a 4-point ordinal response scale: 1 (never), 2 (sometimes), 3 (mostly), or 4 (always), with a “not applicable” response option [16]. The final CanSMART self-management tasks questionnaire comprised 12 items (Additional file 1: Appendix 1, see also Table 2 but note that item on employment is not listed in Table). The team reviewed all materials with an eye to using plain language.

### Recruitment of study sample

A cross-sectional survey was launched in English and French in August 2015 across Canada [9]. We recruited a convenience sample by asking CanSMART team members and representatives of national and regional patient and disease-specific advocacy groups to circulate the survey link via email, newsletters, social media and relevant websites. Advocacy groups were identified by team members and through web searches. In total, 30 advocacy groups were contacted to share study information and the survey link. Interested individuals aged 18 and over, with at least one chronic physical and/or emotional condition were invited to participate. The invitation specified, “To be in this study, you must be 18 years of age, living in Canada, and have one or more chronic physical and/or emotional illnesses”. Age, sex, level of education, employment status, province of residence and type of chronic conditions were included in the brief demographics section appearing at the end of the survey. The online survey did not require any identifying information, and no incentive or compensation was provided. The welcome page informed participants that the survey was voluntary, anonymous, and that completion of the survey was interpreted as informed consent. The study protocol was approved by the Research Ethics Committee of St. Mary’s Hospital Center, Montreal. The survey was closed in February 2016.

### Psychometric analysis

We based the scale development on standard psychometric methodology [19], applied to the ordinal items on self-management task frequency (Additional file 1: Appendix A). The item #12 on managing work-related limitations was excluded, because 57% of the sample had “missing” responses. The study sample was restricted to the subset of patients who had completed the remaining 11 items. The first step in our psychometric analysis was to identify valid subscales by using both Exploratory Factor Analysis (EFA) and Confirmatory Factor Analysis (CFA). Ideally, we would need to work with two large independently collected data sets, and perform scale development on one, using EFA, and validation on the other, using CFA. However, in clinical settings such an approach may not be possible, due to the inherent difficulties of human data collection. Using guidelines recommended by Anderson [20], we therefore randomly split the sample into two equal size subsamples called ‘exploratory’ and ‘confirmatory’. We applied EFA to the former and CFA with maximum likelihood (ML) to the latter [21, 22]. Pearson’s Chi-square and T-tests were used to compare patient characteristics and frequency of self-management tasks in the two subsamples. From the EFA we determined the dimensionality of the factor space, which we refer to as ‘p’; the dimensionality was determined by parallel analysis [22, 23]. To interpret the dimensions found, we listed the ‘important’ items for each dimension, i.e., those with factor loadings (after ‘varimax’) larger than 0.4 [23].

The ‘important’ items for each dimension were grouped to represent one structural equation model (SEM). To take into account variables which we considered meaningful but do not appear ‘important’, we defined informally a number of alternative models, i.e. we consider several ways to assign the non-important variables to any dimension, including removal. We then evaluated these models by CFA. In CFA, several statistics are available to assess the fit of a model. Following Kline [24], we relied on the following four: Chi-Square, Root Mean Square Error of Approximation (RMSEA), Bentler’s Comparative Fit Index (CFI), and the Standardized Root Mean Square Residual (SRMR). Values of Chi-Square close to zero indicate an acceptable model fit; in practice, it is usually required that RMSEA ≤ 0.10, CFI ≥ 0.90, and SRMR ≤ 0.10. Recent references suggest stricter criteria: RMSEA ≤ 0.06, CFI ≥ 0.95, and SRMR ≤ 0.08), but these stricter criteria are only valid for larger sample sizes than ours (n > 500) [25]. In large sample sizes (> 300), since CFA requires (approximate) multivariate normality of the items, ordinal items such as ours might be analyzed by replacing the Pearson correlation matrix with the polychoric correlation matrix [25]; this was not appropriate given our smaller sample size.

The second step in this psychometric analysis was to perform Rasch analysis [26] on the selected model. Likelihood Ratio (LR) tests [27] were performed to determine whether the Partial Credit Model (PCM) [28] or the Rating Scale Model (RSM) [29] would be used. According to Rasch analysis, threshold points on a response scale should be correctly ordered for each item; e.g. the respondents would consider endorsing 'always' to represent greater need for the latent trait rather than 'mostly'. In contrast, ‘disordered thresholds’ occur when respondents have difficulty consistently discriminating between response categories. The Category Characteristic Curve (CCC) [30], which displays the probability of a respondent endorsing a particular response category based on their level of need for the item (intensity), was obtained to examine the performance of the response scale. A disorder threshold was detected in the CCC plot when the line of a corresponding answer crossed other lines (answer) at the same latent trait value; if an item had a disordered threshold, the CCC informed response re-categorisation. To determine item fit, ‘infit’ and ‘outfit’ statistics were calculated, whereby values between 0.7 and 1.3 are acceptable [31]. Finally, unidimensionality was assessed by the Martin-Löf Test [32].

Once validity and ‘scoring properties’ were confirmed, we computed a subscale score for each dimension by simply averaging the important items for the dimension. Internal consistency was assessed using Cronbach’s alpha for each subscale; acceptable reliability can be achieved with a Cronbach Alpha of 0.7 but a threshold of 0.6 is also considered acceptable for short scales (< 10 items) [33].

Finally, we studied the variation of the subscales (computed after Rasch analysis) across subgroups defined by patient characteristics (including diseases). To determine whether these associations were independent we conducted multivariable linear regression analyses [34]. All the analyses were performed with SAS version 9.4 and R. [27] (eRm package).

## Results

### Study sample

The online questionnaire was activated by 353 potential respondents. Of these, 19 entered no information and 28 did not complete the section on self-management tasks. Table 1 presents selected characteristics of the study sample. A large number of the remaining 306 participants did not complete the sociodemographic section of the survey (n = 45). Participants were mostly aged 45 or older (73%), female (78%), and well educated (74% had some college education), spoke English at home (90%), and were born in Canada (87%). Participants reported a mean of 2.8 (SD = 1.6) chronic diseases, with the most common ones being arthritis (62%) and emotional problems (58%).

**Table 1:** Patient Characteristics in the 2 Study Samples (n = 306)

Variables		Overall (n = 306)		Sample EFA (n = 153)		Sample CFA (n = 153)		Chi-square
		n	%	n	%	n	%	*p*-value
Demographics and health section missing		45	14.7	21	13.7	24	15.7	0.628
Demographics		(n = 261)		(n = 132)		(n = 129)		
Age	18–44	71	27	39	30	32	25	0.012
	45–64	124	48	70	53	54	42	
	65–74	66	25	23	17	43	33	
Sex**	Female	198	78	102	80	96	76	0.427
Education**	Secondary school or less	67	26	34	27	33	26	0.946
	College or University	187	74	94	73	93	74	
Geographical location**	City	136	53	65	50	71	56	0.662
	Suburbs of a city	57	22	31	24	26	20	
	Small town or rural	65	25	34	26	31	24	
Born in Canada**	Yes	221	87	113	88	108	85	0.446
Language spoken	English	236	90	119	90	117	91	0.881
	French	25	10	13	10	12	9	
Employment	Currently employed	79	30	46	35	33	26	0.172
	Employment disability	92	35	45	34	47	36	
	Retired	66	25	27	20	39	30	
	Unemployed	24	9	14	11	10	8	
*Health*								
Multimorbidity** (1–19)	Mean (SD)	2.8 (1.6)		2.8 (1.7)		2.7 (1.5)		0.909*
Disease**	Arthritis	157	62	82	64	75	60	0.559
	Emotional problems	147	58	78	61	69	55	0.396
	Hypertension	64	25	35	27	29	23	0.471
	Asthma	55	22	33	26	22	18	0.123
	Neurological	57	22	26	20	31	25	0.375
	Diabetes	40	16	18	14	22	18	0.425
	Autoimmune	39	15	18	14	21	17	0.529
	Heart disease	29	11	11	9	18	14	0.141
	Gastrointestinal	29	11	19	15	10	8	0.092
	Other musculoskeletal	21	8	8	6	13	10	0.225

*T-test; EFA: Exploratory Factor Analysis; CFA: Confirmatory Factor Analysis

**The number of missing values ranges between 3 and 8

### Psychometric results

#### Factor analysis

From the comparison of two subsamples used for EFA and CFA, respectively, we found that only age differed significantly (*p*-value < 0.05) across samples (Table 1). NA (not applicable) responses to the 11 items used in the scale development process were infrequent (mean number of NA was 6 per item, range: 1–19), and were assigned to the response ‘no need’. Only 2 items revealed some departure from normality: “Taking medication daily” (skewness = − 2.2, kurtosis = 3.4) and “Asking for or needing help with self-care” (kurtosis = 2.9): these values are slightly out of the acceptable range (− 2 to + 2 for both) [35].

In the EFA, 2 dimensions were enough to explain the total variance (76% was explained by the first dimension and 23% by the second), see Additional file 1: Appendix 2. The factor loadings (for each item are presented in Table 2; the ‘important’ factor loadings (> 0.4) are in bold font. Three items (Manage discomfort, Take medication daily, Check things such as blood pressure or blood sugar) had factor loadings below threshold for both dimensions, which led us to consider 5 alternative models (corresponding to different ways of dropping items or assigning them to dimensions).

**Table 2:** Exploratory factor analysis on 'exploratory' sample (n = 153)

Items	Dimension #1	Dimension #2
Emotional coping	**0.68**	− 0.04
Managing fatigue	**0.64**	0.18
Avoiding/limiting activities	**0.62**	0.25
Dealing with unexpected or new life problems	**0.52**	0.18
Making lifestyle changes (e.g. diet)	**0.42**	0.15
Asking for or needing help with household chores	0.36	**0.72**
Asking for or needing help with self-care (e.g. bathing)	0.09	**0.68**
Dealing with physical limitations	0.16	**0.50**
Managing discomfort/pain	0.32	0.29
Taking medication daily	0.29	0.08
Monitoring biological parameters (e.g. sugar)	0.03	0.26

Factor loadings after VARIMAX rotation. The important factor loadings are in bold font

CFA was performed on the confirmatory sample to test these five models. The results of CFA are summarized in Table 3, which shows the standardized coefficients of the equations expressing factor estimates as linear combinations of items. According to the Chi-square test, none of the models fits the data well (*p*-value < 0.05), but other indices suggested acceptable fits. Only Model 1 showed an acceptable fit for all other indices. We therefore retained Model 1; the two subscales were labeled as: a) *Coping tasks* (Managing discomfort/pain, Managing fatigue, Making lifestyle changes (e.g. diet), Emotional coping, Avoiding/limiting activities, Dealing with unexpected or new life problems), and b) *Physical tasks* (Dealing with physical limitations, Asking for or needing help with household chores, Asking for or needing help with self-care).

**Table 3:** Confirmatory Factor Analysis with the 'Confirmatory' Sample (best models) (n = 153)

	Scenarios									
	Model 1		Model 2		Model 3		Model 4		Model 5	
	Dim #1	Dim #2	Dim #1	Dim #2	Dim #1	Dim #2	Dim #1	Dim #2	Dim #1	Dim #2
*Items*										
Emotional coping	0.39	–	0.39	–	0.41	–	0.39	–	0.39	–
Managing fatigue	0.61	–	0.61	–	0.62	–	0.61	–	0.61	–
Avoiding/limiting activities	0.70	–	0.70	–	0.68	–	0.70	–	0.70	–
Dealing with unexpected or new life problems	0.50	–	0.50	–	0.54	–	0.52	–	0.52	–
Making lifestyle changes (e.g. diet)	0.38	–	0.38	–	0.40	–	0.39	–	0.39	–
Asking for or needing help with household chores	–	0.79	–	0.80	–	0.81	–	0.74	–	0.74
Asking for or needing help with self-care (e.g. bathing)	–	0.55	–	0.55	–	0.54	–	0.56	–	0.56
Dealing with physical limitations	–	0.59	–	0.59		0.58	–	0.59	–	0.59
Managing discomfort/pain	0.53	–	0.53	–	–	–	–	0.50	–	0.50
Taking medication daily	–	–	0.10*	–	–	–	–	–	–	–
Monitoring biological parameters (e.g. sugar)	–	–	–	0.06*	–	–	–	–	–	0.05*
*Measures of fit*										
Chi-square test (value, *df*, *p*-value)	Value = 52.7, *df* = 26, *p* = 0.002		Value = 73.7, *df* = 43, *p* = 0.003		Value = 46.4, *df* = 19, *p* < 0.001		Value = 58.7, *df* = 26, *p* < 0.001		Value = 68.0, *df* = 34, *p* < 0.001	
Root Mean Square Residual (RMSEA)	0.08 [0.05; 0.11]		0.07 [0.04; 0.10]		0.10 [0.06; 0.13]		0.09 [0.06; 0.12]		0.08 [0.05; 0.11]	
Standardized Root Mean square Residual (SRMR)	0.07		0.07		0.08		0.08		0.08	
Bentler's Comparative Fit Index (CFI)	0.90		0.88		0.88		0.87		0.87	
*Internal consistency*										
Cronbach's alpha (standardized)	0.70	0.67	0.65	0.54	0.67	0.67	0.67	0.68	0.67	0.59

*All the standardized effects are significant at alpha 0.05, except those with an asterisk (Dim: Dimension)

#### Rasch analysis

The PCM fit the data better than did the RSM for the *Coping tasks* (LR = 18, *df* = 5, *p*-value < 0.001), but not for the *Physical tasks* (LR = 3.15, *df* = 2, *p* = 0.207). Despite this*,* we report the results for the PCM, since the findings were the same as the RSM (PCM can handle items with different scaling).

All items show a potential ‘disorder’ in the region from ‘Mostly’ to ‘Always’. Also, Additional file 1: Appendix 3a and 3c show that the line corresponding to the answer ‘Sometimes’ crosses the other two lines at the same latent trait value. This inspection suggested recoding ‘Mostly’ and ‘Always’ as the same response. Indeed, the new CCC graphs did not suggest ‘disorder’ (see Additional file 1: Appendix 3b and 3d), except for the item “Asking for or needing help with self-care” (*Physical Tasks* subscale); in this case the CCC plot suggest grouping ‘Sometimes’ with ‘Mostly and Always’.

After these changes, the mean square fit statistics for all items were acceptable, ‘infit’ and ‘outfit’ ranging from 0.69 to 1.00 (details not shown). The Martin–Löf Test for unidimensionality for *Coping tasks* is consistent with one dimension (*p*-value = 0.769); this test was not performed for the 3 items *Physical tasks* subscale since it requires at least 4 items.

#### Cronbach’s alpha

The Cronbach’s alphas for Model 1 were 0.70 for *Coping tasks*, and 0.67 for *Physical tasks.*

#### Associations with patient characteristics

Table 4 shows the results of the regression of each subscale on patient characteristics. Note that the self-management tasks subscales were computed using the scoring suggested by the Rasch analysis (see Table 5): all items except one used a 3-level response scale: never, sometimes, mostly/always. One item (asking for or needing help with self-care) used the binary scoring suggested by the Rasch analysis: never and sometimes/mostly/always. Mean frequencies of both *Coping and Physical tasks* were significantly higher among participants on employment disability, and among those with musculoskeletal conditions other than arthritis. Also, the mean frequency of *Coping tasks* was significantly higher among female participants, those with emotional conditions, asthma, and no heart disease. The mean frequency of *Physical tasks* was significantly higher among participants with arthritis, and neurological conditions. The regression analyses presented in Table 4 that includes the number of diseases and not the individual diseases (not shown) revealed similar significant results for all variables except for the Physical subscale, whereby the effect of age was significant (older patient have more tasks); the effect of the number of diseases was significant, having 1 more disease is associated with a 3% increase in both scores. Of the two medical management items that did not load on either subscale, both were significantly associated with employment disability (not shown in the Tables). The medication task frequency item was significantly higher among participants who reported emotional problems; the self-monitoring of biological parameters was associated significantly with a diagnosis of diabetes and hypertension.

**Table 4:** Association between patient characteristics and self-management tasks subscales* (coping and physical), results of multivariable linear regression models (n = 246**)

Variables		Coping tasks		Physical tasks	
		Beta	95% CI	Beta	95% CI
*Demographics*					
Age	18–44	0.00		0.00	
	45–64	0.01	[**−** 0.10; 0.11]	0.14	[**−** 0.01; 0.28]
	65–74	0.14	[**−** 0.04; 0.32]	0.20	[**−** 0.04; 0.44]
Sex	Male	**− 0.18**	**[−** **0.29; −** **0.08]**	**−** 0.02	[**−** 0.16; 0.12]
Education	Secondary school or less (ref: college or university)	0.07	[**−** 0.03; 0.16]	0.09	[**−** 0.04; 0.21]
Geographical location	City	0.00		0.00	
	Suburbs of a city	**−** 0.04	[**−** 0.13; 0.06]	0.04	[**−** 0.09; 0.18]
	Small town or rural	**−** 0.04	[**−** 0.14; 0.05]	0.00	[**−** 0.13; 0.14]
Language spoken	French (ref: English)	0.08	[**−** 0.08; 0.23]	0.11	[**−** 0.11; 0.32]
Employment	Currently employed	0.00		0.00	
	Employment disability	**0.13**	**[0.03; 0.23]**	**0.23**	**[0.10; 0.37]**
	Retired	**−** **0.19**	**[−** **0.35; −** **0.04]**	**−** 0.08	[**−** 0.29; 0.13]
	Unemployed	**−** 0.03	[**−** 0.18; 0.12]	**−** 0.11	[**−** 0.31; 0.10]
*Chronic diseases*					
Arthritis	Yes	0.07	[**−** 0.02; 0.15]	**0.24**	**[0.13; 0.36]**
Emotional problems	Yes	**0.14**	**[0.05; 0.22]**	**−** 0.02	[**−** 0.13; 0.09]
Hypertension	Yes	0.10	[**−** 0.00; 0.22]	0.07	[**−** 0.06; 0.21]
Asthma	Yes	**0.11**	**[0.01; 0.22]**	0.06	[**−** 0.08; 0.20]
Neurological	Yes	0.07	[**−** 0.03; 0.17]	**0.23**	**[0.09; 0.37]**
Diabetes	Yes	0.01	[**−** 0.11; 0.13]	0.07	[**−** 0.10; 0.23]
Autoimmune	Yes	0.09	[**−** 0.03; 0.20]	0.07	[**−** 0.09; 0.22]
Heart disease	Yes	**−** **0.15**	**[−** **0.29; −** **0.02]**	**−** 0.03	[**−** 0.22; 0.15]
Gastrointestinal	Yes	0.04	[**−** 0.09; 0.17]	**−** 0.05	[**−** 0.22; 0.13]
Other musculoskeletal	Yes	**0.15**	**[0.01; 0.30]**	**0.20**	**[0.01; 0.39]**

Linear regression, significant (*p* < 0.05) beta estimates are in bold font

*Coding based on system shown in Table 5

**n = 261 but we have missing values for diseases, education and sex, the final study sample is n = 246

Including the number of diseases instead of the individual diseases revealed similar significant results except for the Physical subscale, whereby the effect of age was significant (older patient have more tasks); the effect of the number of diseases was significant: having 1 more disease is associated with a 3% increase in both scores

**Table 5:** Self-management tasks frequency by subscales

Scales and questions			
How often you do them to manage your illness(es)?	Responses (number of points)		
*Coping tasks*			
(1) Deal with feelings of worry or sadness or fears that affect any or all of your daily life, including family and work relationships	Never (1)	Sometimes (2)	Mostly or Always (3)
(2) Allow for extreme tiredness or limited energy when planning your day	Never (1)	Sometimes (2)	Mostly or Always (3)
(3) Avoid/limit activities that you enjoy doing (e.g., social activities with family/friends, doing hobbies)	Never (1)	Sometimes (2)	Mostly or Always (3)
(4) Deal with unexpected or new problems in your life and/or work due to your illness (e.g., financial issues; changes in illness or a new illness)	Never (1)	Sometimes (2)	Mostly or Always (3)
(5) Make changes to any part of your diet (food or fluids), your activities, or your medications due to a change in your illness	Never (1)	Sometimes (2)	Mostly or Always (3)
(6) Managing discomfort and pain	Never (1)	Sometimes (2)	Mostly or Always (3)
*Physical tasks*			
(1) Ask for or need help with household chores (e.g., preparing meals, cleaning your home, doing laundry, grocery shopping)	Never (1)	Sometimes (2)	Mostly or Always (3)
(2) Ask for or help with self-care (e.g. eating, dressing, bathing)*	Never (1)	Sometimes, Mostly or Always (2)	
(3) Dealing with physical limitations	Never (1)	Sometimes (2)	Mostly or Always (3)
*Miscellaneous (all coded initially as never, sometimes, mostly, always)*			
(1) Check things such as blood pressure or blood sugar levels			
(2) Take medications daily			
(3) (if you are working): Miss work, have to leave early, work shorter hours or struggle to complete all your work			

*Binary coding as suggested by the Rasch analysis. Alternatively, the same 3 category coding as other items can be used, as per sensitivity analyses

We also conducted a sensitivity analysis, not presented here, in which all items were coded using the same 3-level response scale. The results were almost identical to those reported in Table 4. On average the beta coefficient varied by ± 5%, with the exception of age 65–74 years (20% variation). None of these variations led to changes in the statistical significance, with the exception of musculoskeletal diseases which became statistically non-significant (beta: 0.18 [− 0.02; 0.38]).

Table 5 shows the final self-management task subscales with response options and scoring based on the Rasch analysis.

## Discussion

This study sought to advance chronic disease self-management research through the collaborative development with patient partners of a measure to assess the type and frequency of self-management tasks and to conduct a preliminary analysis of its psychometric properties.

Using accepted guidelines on scale development, we randomly separated the sample into two subsamples of equal size and used one subsample for EFA and the other for CFA, a major step towards valid scale development. Our psychometric analysis revealed two self-management task subscales: one assessing frequency of *Coping tasks* (Managing discomfort/pain, Managing fatigue, Making lifestyle changes (e.g. diet), Emotional coping, Avoiding/limiting activities, Dealing with unexpected or new life problems), and the second assessing *Physical tasks* (Dealing with physical limitations, Asking for or needing help with household chores, Asking for or needing help with self-care). The Rasch analyses suggested appropriate response scales for each item. Two additional tasks (medication use and self-monitoring of biological parameters) did not load on either subscale and may represent additional dimensions of self-management.

There has been little prior quantitative research on describing the tasks that people with chronic diseases do—the work of self-management. The subscales developed in the CanSMART study do not map exactly onto the categories identified in the qualitative synthesis of Schulman–Green [15], although they include many of the processes described. Our Coping self-management tasks subscale captures several aspects of the third category (adjusting, integrating) along with health behaviors, which could also be conceptualized as coping activities. Our Physical subscale captures aspects of the second category dealing with mobilizing help for daily activities. Illness needs were represented in the CanSMART survey by items on taking medication and on self-monitoring. Neither of these items loaded onto our two subscales. In the case of taking medication, this task is very frequent among most people with chronic illnesses and represents an important dimension of self-management. In the case of self-monitoring, the need for this task and the type of monitoring required appeared to be quite disease-dependent, being performed more frequently for hypertension (blood pressure monitoring) and diabetes (blood sugar monitoring). These two illnesses have established measurement tools and treatment benchmarks—which give patients discrete targets to aim for, and can make self-monitoring more straightforward. It should be noted that the item wording may have biased respondents to reporting these two types of self-monitoring rather than others (e.g., self-monitoring of depression symptoms). Similar to our results, Schulman-Green [15] also reported that comorbidities increased the type and complexity of self-management tasks performed.

Van Houtum et al. [36] used a theory-based approach in developing the PAST scales of self-management tasks and related support needs. In contrast, in CanSMART, we asked patients to review items and adapted them as needed to ensure that they were understandable and relevant. The PAST scales distinguished four types of self-management tasks: medical management (including medication management and self-monitoring); communication with healthcare providers; coping with the consequences of disease; and making lifestyle changes. Again, our Coping self-management tasks subscale overlaps substantially with the PAST coping scale. Lifestyle changes were covered in a single question in CanSMART. As noted above, medical management did not emerge as a distinct dimension. Communication with healthcare providers was assessed in a separate scale, the PACIC [17]. This is an important aspect of self-management and should be considered for inclusion in future iterations for the CanSMART questionnaire. We separated coping with pain and physical limitations into two different items, and in fact these items loaded into two different factors: pain with coping tasks, and physical limitations with physical tasks.

One CanSMART question considered important by our patient partners addressed employment issues such as absenteeism; however, this item could not be included in the psychometric analysis because of missing data for a significant proportion of participants. Nevertheless, our secondary analysis suggested that this item correlated in particular with the Coping task subscale.

The validity of the task subscales and items is supported by their associations with patient characteristics For example, the Coping tasks subscale was associated with employment disability and certain diagnoses (emotional, asthma, and other musculoskeletal). Women reported higher overall coping task frequency than men. The Physical self-management tasks subscale was associated with employment disability, arthritis, neurological, and other musculoskeletal diseases. The medication task frequency item was significantly higher among participants with employment disability, and those who reported emotional problems; the self-monitoring of biological parameters was associated significantly with a diagnosis of diabetes and hypertension, both diseases commonly requiring patient self-monitoring [37].

The Coping and Physical subscales, when finalized, have multiple applications in research and practice. For example, they could be used to target interventions. Our previous research using these scales found that they could be used to identify vulnerable self-managers, with high frequency of self-management tasks but low self-efficacy in performing them [9].

## Limitations

The CFA was performed on a sample of 153 participants. In view of our limited sample size, we did not perform more complex analysis, such as maximum likelihood with robust standard error and weighted least square with polychoric correlation. It would be desirable to repeat the CFA on a larger sample size (at least 300 is recommended [38]). As regards the Rasch analysis, participants had difficulty discriminating between categories “mostly” and “always”, suggesting that these categories should be merged in future studies to calculate the subscale scores. Only one item needed a different re-coding (collapsing “sometimes”, “mostly” and “always”). This difference might limit the practical use of the scale. However, our sensitivity analyses using uniform three category coding revealed almost identical results suggesting that universal application of the 3-level response set would be acceptable.

Finally, the sample is one of convenience and results may not generalize to all Canadian adults with chronic diseases. In particular, the sample is more highly educated than the general population. Future studies might want to consider quota sampling to ensure enough participants are of lower education status and are still employed to further assess the psychometric properties of the scale.

## Conclusion

Our study is one of the first to attempt to measure quantitatively the tasks of chronic disease self-management. As such, it provides a preliminary basis on which further scale development and psychometric analysis could be conducted. Our study confirms the existence of a distinct coping dimension of self-management, in keeping with previous research. Our results also suggest a second distinct dimension that addresses mobilization of family and community resources to assist with physical self-care. Questions on mobilization of medical professionals should be added to future versions. Among the other items measured, our results suggest that medication management is an almost universal task. On the other hand, self-monitoring appears to be disease specific.

## Supplementary Information

**Additional file 1**. Appendices.

## Data Availability

The datasets used and/or analysed during the current study available from the corresponding author on reasonable request.

## Abbreviations

CanSMART: Canadian self-management research team
CCC: Category characteristic curve
CFA: Confirmatory factor analysis
CFI: Comparative fit index
EFA: Exploratory factor analysis
LR: Likelihood ratio
ML: Maximum likelihood
PACIC: Patient assessment of chronic illness care
PAST: Dutch patient assessment of self-management tasks
PCM: Partial credit model
RMSEA: Root mean square error of approximation
RSM: Rating scale model
SD: Standard deviation
SEM: Structural equation model
SRMR: Standardized root mean square residual
