# Supplementary material for: Development and psychometric evaluation of the CanSmart questionnaire to measure chronic disease self-management tasks
Source: BMC Psychol. 2022 Dec 7;10:293. doi: 10.1186/s40359-022-00995-2 (PMC9730574; doi:10.1186/s40359-022-00995-2)

**Appendix 1.** Self-Management Task Frequency: Survey Items

Below is a list of common things that people who live with one or more chronic illnesses have to deal with on a regular basis. Please tell us how often you do them to manage your illness(es).

| **Because of your illness(es), do you need to…** | **Never** | **Sometimes** | **Mostly** | **Always** | **Not applicable** |
|---|---|---|---|---|---|
| 1. Manage discomfort and pain | ☐ | ☐ | ☐ | ☐ | ☐ |
| 2. Deal with physical limitations (mobility) | ☐ | ☐ | ☐ | ☐ | ☐ |
| 3. Take medications daily | ☐ | ☐ | ☐ | ☐ | ☐ |
| 4. Make changes to any part of your diet (food or fluids), your activities, or your medications due to a change in your illness | ☐ | ☐ | ☐ | ☐ | ☐ |
| 5. Allow for extreme tiredness or limited energy when planning your day | ☐ | ☐ | ☐ | ☐ | ☐ |
| 6. Deal with feelings of worry or sadness or fears that affect any or all of your daily life, including family and work relationships | ☐ | ☐ | ☐ | ☐ | ☐ |
| 7. Ask for or need help with self-care (e.g. eating, dressing, bathing, toileting) | ☐ | ☐ | ☐ | ☐ | ☐ |
| 8. Ask for or need help with household chores (e.g., preparing meals, cleaning your home, doing laundry, grocery shopping) | ☐ | ☐ | ☐ | ☐ | ☐ |
| 9. Avoid / limit activities that you enjoy doing (e.g., social activities with family/friends, doing hobbies) | ☐ | ☐ | ☐ | ☐ | ☐ |
| 10. Check things such as blood pressure or blood sugar levels.<br>Please list the things you have to check: | ☐ | ☐ | ☐ | ☐ | ☐ |
| 11. Deal with unexpected or new problems in your life and/or work due to your illness (e.g., financial issues; changes in illness or a new illness) | ☐ | ☐ | ☐ | ☐ | ☐ |
| 12. (if you are working): Miss work, have to leave early, work shorter hours or struggle to complete all your work | ☐ | ☐ | ☐ | ☐ | ☐ |

**Appendix 2.** Parallel Analysis Median Simulated Eigenvalues
(11 variables, 1000 iterations and n=153 observations)

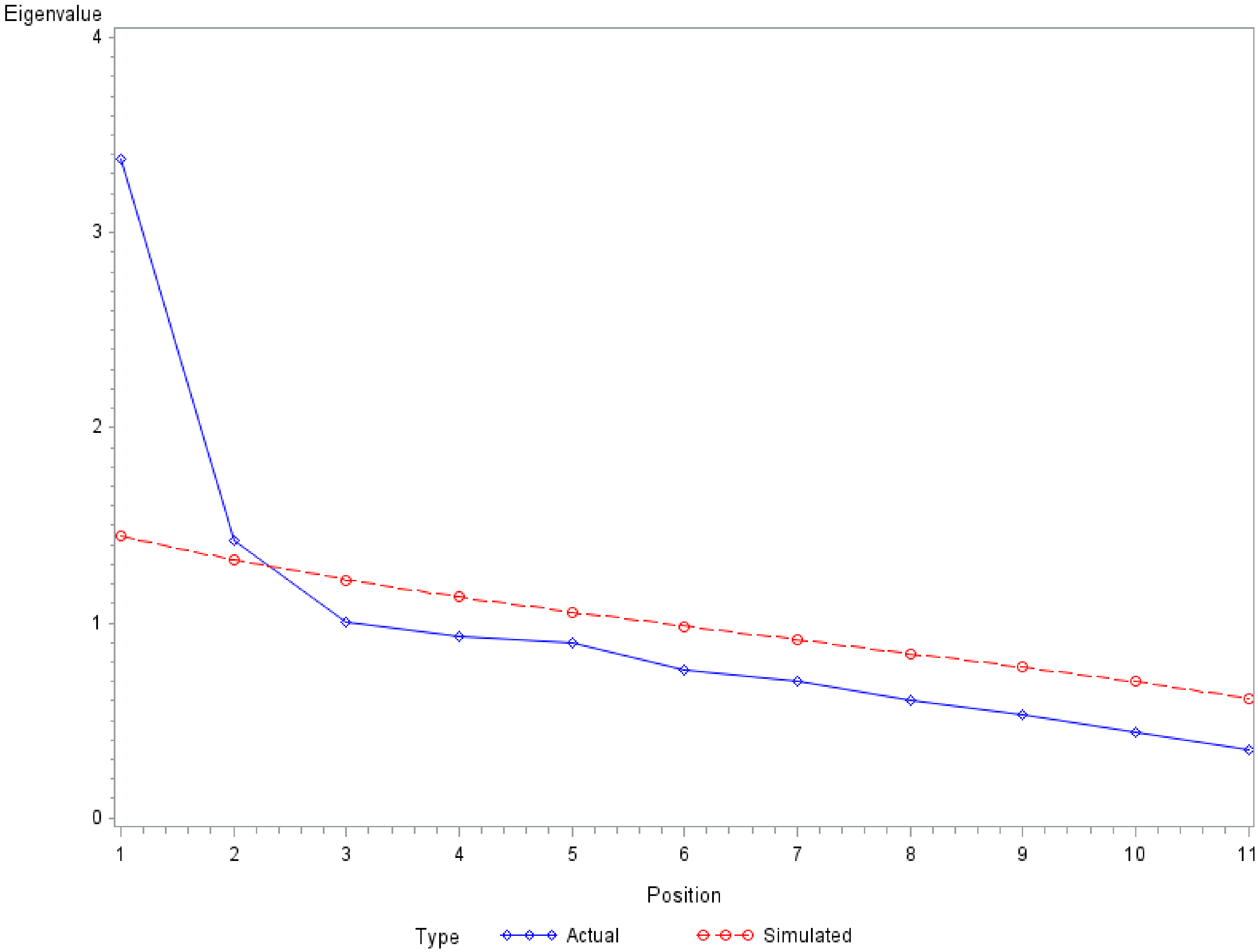


**Interpretation.** The intersection of the two lines (observed (actual) and empirical (simulated)), show that the number of dimensions is closer to 2.

**Appendix 3a.** Category Characteristic Curve (CCC) for an Item for Each Item (coping scale)
[Black: Never, Red: Sometimes, Green: Mostly, Blue: Always]

Managing pain

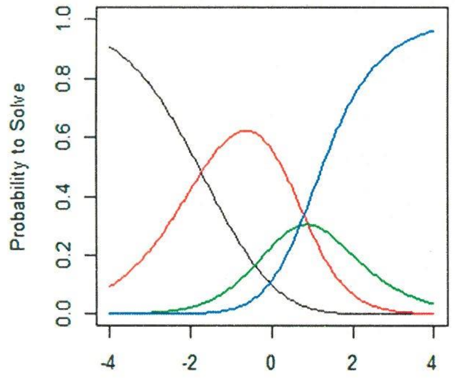


Lifetime changes

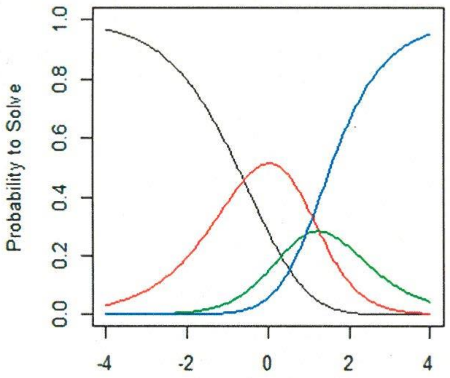


Managing fatigue

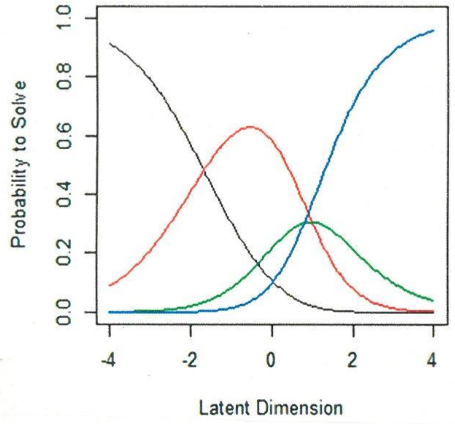


Emotional coping

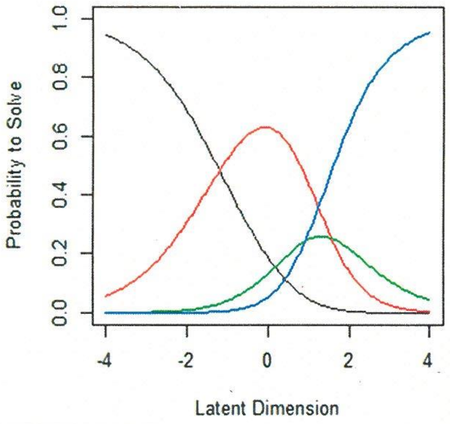


Avoiding activities

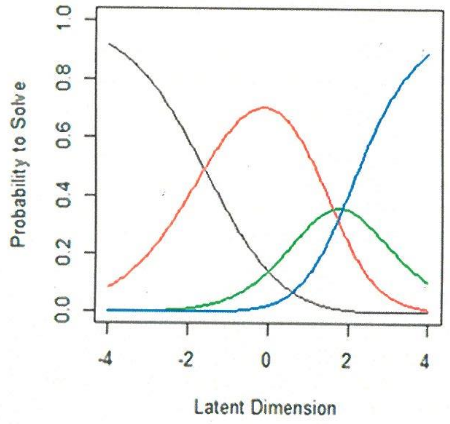


New life problems

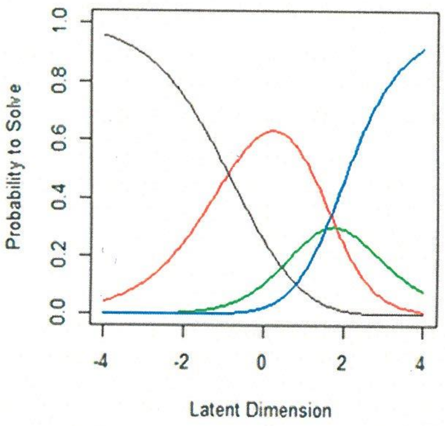

**Appendix 3b.** Category Characteristic Curve (CCC) for an Item for Each Item (coping scale)
[Black: Never, Red: Sometimes, Green: Mostly or Always]

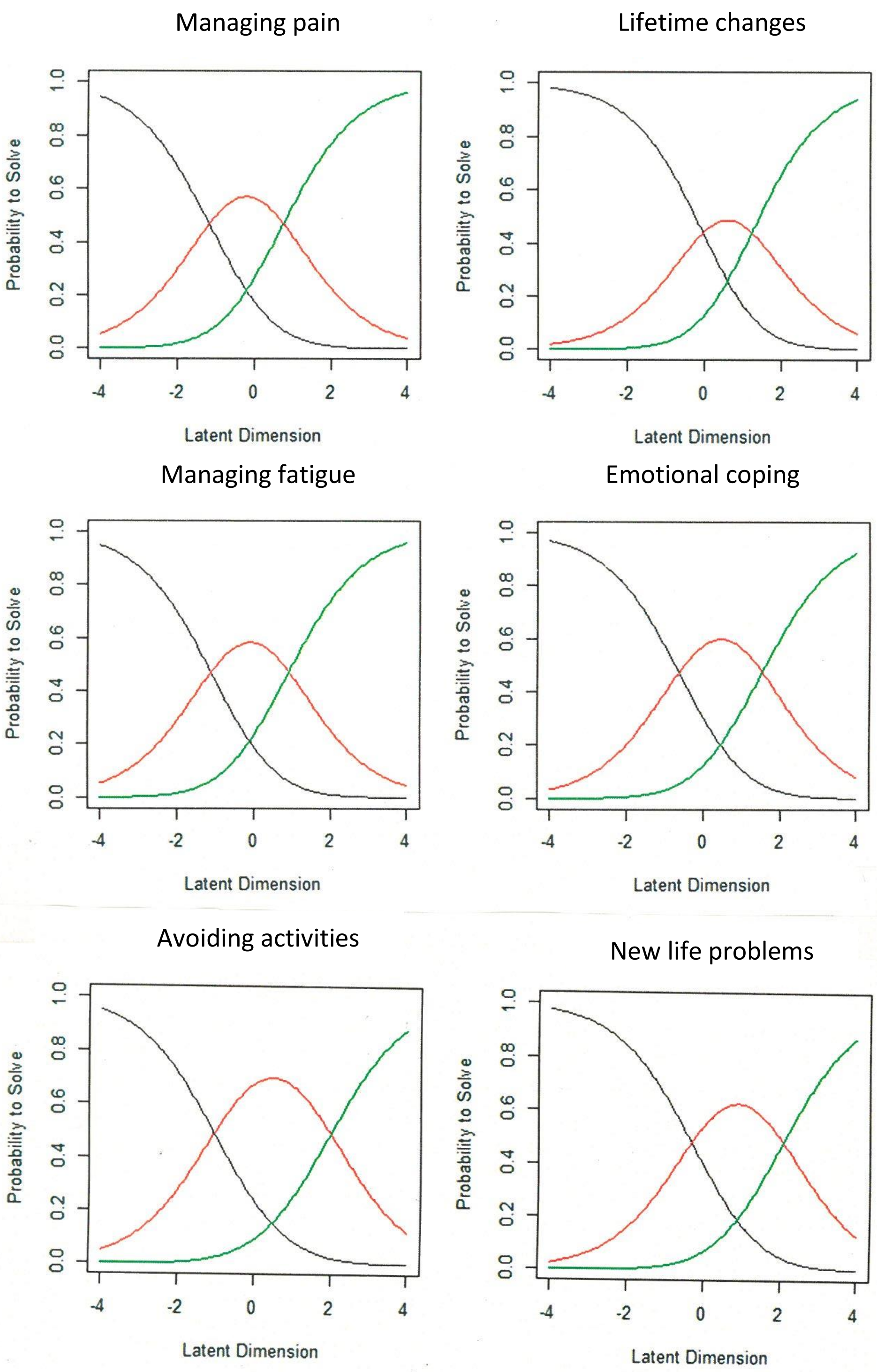

**Appendix 3c.** Category Characteristic Curve (CCC) for an Item for Each Item (physical scale)
[Black: Never, Red: Sometimes, Green: Mostly, Blue: Always]

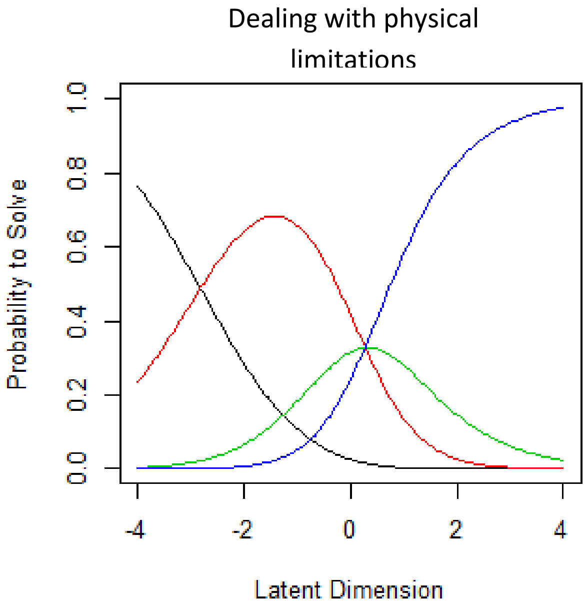


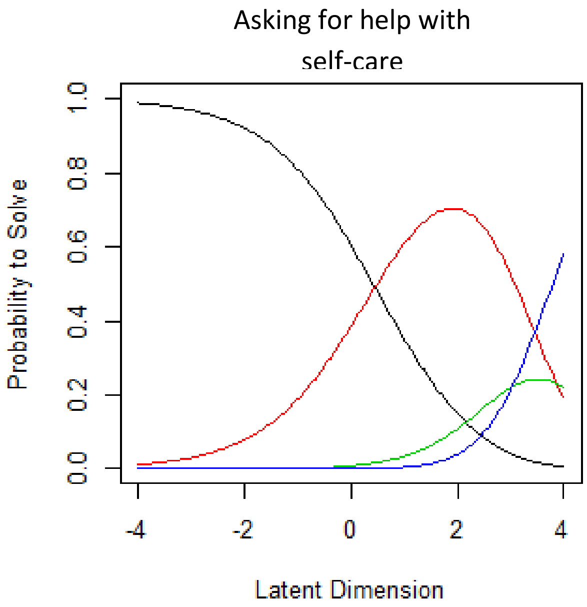


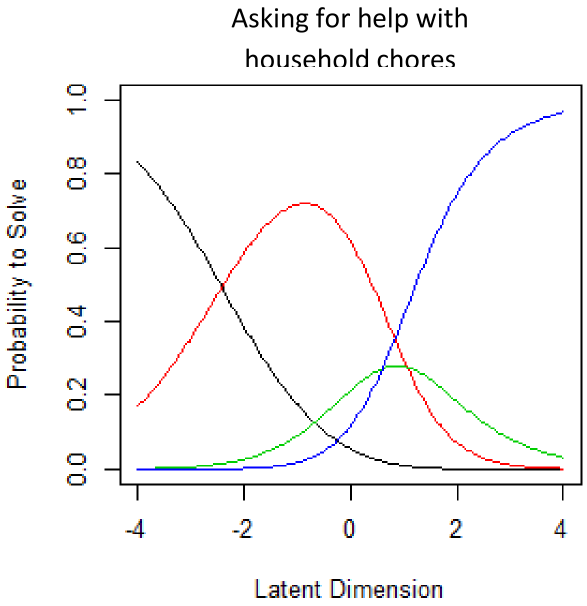

**Appendix 3d.** Category Characteristic Curve (CCC) for an Item for Each Item (physical scale)
[Black: Never, Red: Sometimes, Green: Mostly or Always]

Dealing with physical limitations

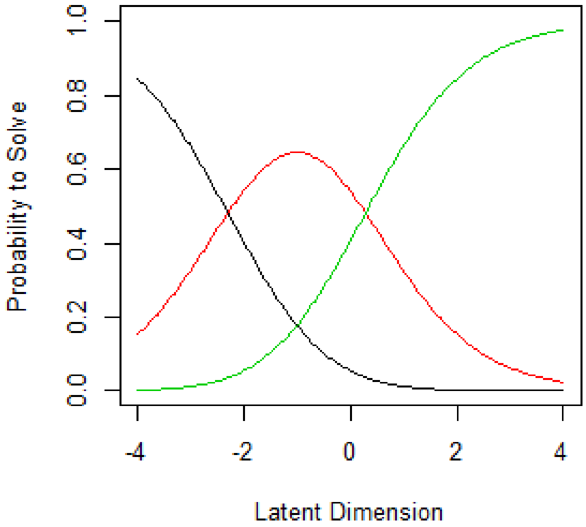


Asking for help with self-care

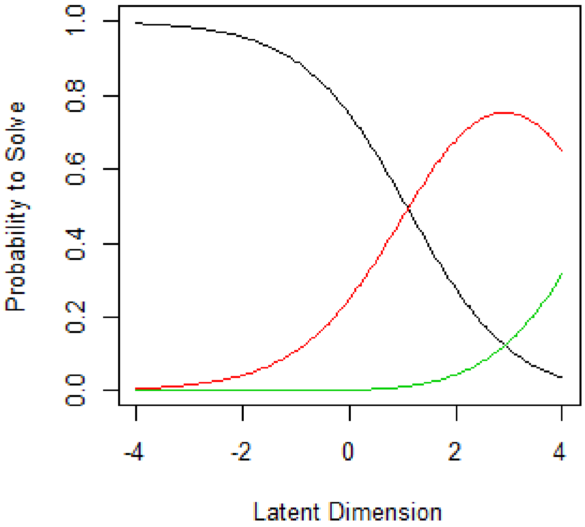


Asking for help with household chores

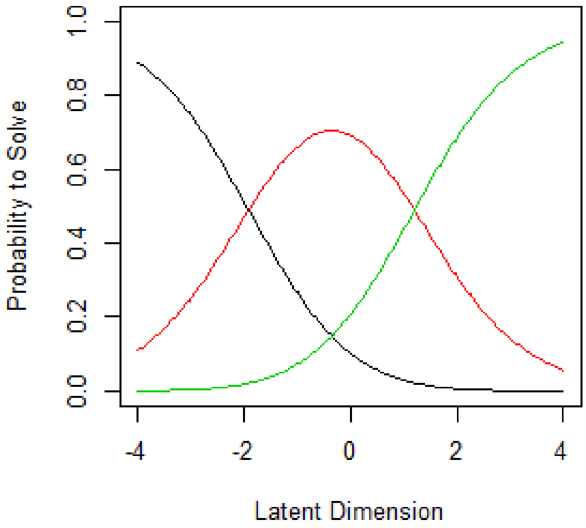

Supplement: Supplementary file 1 — Additional file 1. Appendices. [file 40359_2022_995_MOESM1_ESM.pdf]
